# Case report: Immunovirotherapy as a novel add-on treatment in a patient with thoracic NUT carcinoma

**DOI:** 10.3389/fonc.2022.995744

**Published:** 2022-10-27

**Authors:** Linus D. Kloker, Branko Calukovic, Katrin Benzler, Alexander Golf, Sebastian Böhm, Sven Günther, Marius Horger, Simone Haas, Susanne Berchtold, Julia Beil, Mary E. Carter, Tina Ganzenmueller, Stephan Singer, Abbas Agaimy, Robert Stöhr, Arndt Hartmann, Thomas Duell, Sandra Mairhofer, Fabian Fohrer, Niels Reinmuth, Lars Zender, Ulrich M. Lauer

**Affiliations:** ^1^ Department of Medical Oncology and Pneumology, Virotherapy Center Tübingen (VCT), Medical University Hospital, Tübingen, Germany; ^2^ Diagnostic and Interventional Radiology, University Hospital Tübingen, Tübingen, Germany; ^3^ Max-Planck-Institute of Biochemistry, Department of Molecular Medicine, Martinsried, Germany; ^4^ German Cancer Consortium (DKTK), German Cancer Research Center (DKFZ), Tübingen, Germany; ^5^ Institute for Medical Virology and Epidemiology of Viral Diseases, University Hospital Tübingen, Tübingen, Germany; ^6^ Department of Pathology, University Hospital Tübingen, Tübingen, Germany; ^7^ Department of Pathology, University Hospital Erlangen, Erlangen, Germany; ^8^ Asklepios Lung Clinic, Munich-Gauting, Munich, Germany; ^9^ DFG Cluster of Excellence 2180 ‘Image-guided and Functional Instructed Tumor Therapy’, University of Tübingen, Tübingen, Germany

**Keywords:** NUT carcinoma, virotherapy, immunotherapy, T-VEC, chemotherapy, NUTM1, BRD3

## Abstract

NUT carcinoma (NC) is a rare and extremely aggressive form of cancer, usually presenting with intrathoracic or neck manifestations in adolescents and young adults. With no established standard therapy regimen and a median overall survival of only 6.5 months, there is a huge need for innovative treatment options. As NC is genetically driven by a single aberrant fusion oncoprotein, it is generally characterized by a low tumor mutational burden, thus making it immunologically cold and insusceptible to conventional immunotherapy. Recently, we have demonstrated that oncolytic viruses (OVs) are able to specifically infect and lyse NC cells, thereby turning an immunologically cold tumor microenvironment into a hot one. Here, we report an intensive multimodal treatment approach employing for the first time an OV (talimogene laherparepvec (T-VEC); IMLYGIC^®^) together with the immune checkpoint inhibitor pembrolizumab as an add-on to a basic NC therapy (cytostatic chemotherapy, radiation therapy, epigenetic therapy) in a patient suffering from a large thoracic NC tumor which exhibits an aberrant, unique *BRD3:NUTM1* fusion. This case demonstrates for the first time the feasibility of this innovative add-on immunovirotherapy regimen with a profound, repetitive and durable replication of T-VEC that is instrumental in achieving tumor stabilization and improvement in the patient´s quality of life. Further, a previously unknown *BRD3:NUTM1* fusion gene was discovered that lacks the extraterminal domain of *BRD3*.

## Introduction

### NUT carcinoma

NUT carcinoma (nuclear protein in testis carcinoma, NC), formerly known as NUT midline carcinoma, is a poorly differentiated and highly aggressive tumor which is molecularly defined by an aberrant *NUTM1* fusion gene (1).

This neoplasm can arise in all age groups, but mostly adolescents and young adults are affected with a median onset of disease at 23.6 years (2).

Very rarely, when the diagnosis can be made in a still localized state, surgery and adjuvant radiation can achieve long-term survival (3, 4), particularly when resection achieves tumor-free margins (5). Unfortunately, patients usually present in an advanced metastatic and therefore inoperable condition, facing a fatal prognosis with a median overall survival (OS) of 6.5 months only. Three different prognostic subgroups have been described depending on tumor localization and type of fusion gene, with (i) the best median OS in patients with non-thoracic non-*BRD4:NUTM1* fusion carcinoma (36.5 months from initial cancer diagnosis), (ii) a median prognosis for non-thoracic disease with *BRD4::NUTM1* fusion (10 months) and (iii) the worst prognosis (4.4 months) in patients with thoracic disease onset, independent of the fusion gene partner (2).

The foremost common fusion partner gene is *BRD4* (75%), followed by *BRD3*, *NSD3* and *ZNF532* (6). Frequently, NC is initially misdiagnosed as a poorly differentiated squamous cell carcinoma (SCC), but simple immunostaining for NUT allows a straightforward diagnosis (7). Fluorescence *in situ* hybridization or next generation sequencing approaches, including panel or exome sequencing, can reveal the corresponding fusion partner gene.

So far, there is no standard treatment regimen for palliation of metastatic disease stages. However, ifosfamide-based regimens and additional radiation therapy have shown some promising results, with published cases of some very rare partial or complete responses in non-thoracic NC (6, 8–11). The combination of carboplatin and paclitaxel is frequently used in thoracic NC, but with only limited success and progression free survival rates below 3 months (12–14).

### Oncolytic virotherapy

Oncolytic virotherapy employs oncolytic viruses (OVs) to specifically infect, replicate in and lyse tumor cells whilst sparing healthy cells. Such OVs are specifically selected based on their natural tumor tropism and/or are genetically modified for improved and even more selective tumor tropism. During intratumoral (i.t.) virus replication and subsequent direct virus-mediated oncolysis, tumor neo-antigens as well as pathogen and damage associated molecular patterns (PAMPs and DAMPs) are released, thereby inducing a highly inflamed tumor microenvironment as well as an additional indirect (immunogenic) tumor cell death associated with a powerful systemic anti-tumor immune response (15). Of note, the anti-tumoral efficacy of ICIs was shown to be augmented by intratumoral application of the virotherapeutic compound T-VEC (16, 17). OVs are thought to elicit a response particularly in tumors that have an immunosuppressive tumor microenvironment and a low tumor mutation burden, and thus show very low response rates to immune checkpoint inhibition as monotherapy (18).

### The oncolytic virus T-VEC

T-VEC (talimogene laherparepvec; IMLYGIC^®^) is a genetically engineered oncolytic herpes simplex virus type 1 (HSV-1), modified for specific tumor replication and enhanced immunostimulation, while being attenuated in healthy and especially in brain tissue (19). In 2015, T-VEC was the first and only OV to be approved by the US Food and Drug Administration (FDA), as it showed a significant benefit for treatment of advanced stages of metastatic melanoma (20). However, a recent phase III study (Masterkey-265, NCT02263508) failed to show significant benefits of the combined treatment with T-VEC and the ICI pembrolizumab in melanoma, despite indicating positive trends in the combination group. Currently, this regimen is being evaluated for treatment of melanoma in a neoadjuvant setting (NCT04330430, NCT04427306) as well as for other tumor entities such as sarcomas, triple-negative breast cancer or peritoneal surface malignancies.

For melanoma, T-VEC was already demonstrated to increase the number of tumor infiltrating CD4- and CD8-lymphocytes as well as activated CD8-lympho-cytes in the circulation and to rise expression of PD-L1 and IFNγ in virus-injected tumors (16, 17). Preclinical data suggest an alteration of the tumor microenvironment by increased neutrophils, monocytes and chemokines and the induction of tumor specific T-lymphocytes (21). These features enable T-VEC to turn immunologically cold tumors into a hot ones (22).

### Immunotherapy in NUT carcinoma

So far, virotherapeutics, such as T-VEC, have not been employed in the treatment of NC. Moreover, there are only a few reported cases and no clinical trials for NC treatment with ICIs. Nevertheless, off-label treatment with ICIs is a frequent practice. In a case report, Davis et al. described a patient with thoracic NC for whom treatment with initial surgery, adjuvant chemotherapy and the anti-PD-1 agent nivolumab resulted in long-term survival of 7.5 years. Nivolumab was administered for 3.5 years, starting at disease recurrence three years after initial diagnosis (23). Previous work suggests a low PD-L1 expression and TMB in the majority of NCs (24), which generally predicts a rather poor response rate to sole PD-1 blockade.

In another case series of five NC patients treated with ICIs, the response rate to immunotherapy could be improved by previous radiation therapy, whereas one patient with high TMB did not respond to immunotherapy (12). A further case analysis of twelve patients also supported the hypothesis that radiotherapy followed by immunotherapy results in beneficial outcomes. Regarding the usage of distinct ICIs, best outcomes were seen with pembrolizumab, whereas atezolizumab so far showed no benefit (25).

It remains unclear if TMB and PD-L1 expression as predictive biomarkers are also applicable to NC, since individual differences in NC location and stage at diagnosis might overshadow reliable evidence with the reported small sample sizes. Nevertheless, infiltration of neutrophils and T-cells into NC tissues have been described (24, 26, 27). An additional strong stimulus for attracting more T-cells to the tumor sites, e.g. oncolytic virotherapy may be required and thus is a prerequisite for any effective anti-tumoral immune activation.

### Case highlights

In this patient case, T-VEC is employed for treatment of NC for the first time by sequential intratumoral injections. It is combined with a multimodal treatment approach including chemotherapy, radiotherapy, an HDAC inhibitor and an ICI (pembrolizumab). This patient case demonstrates a treatment response to this multimodal approach as well as repetitively high replication yields of T-VEC when applied intratumorally into different NC locations. Moreover, a previously undescribed *BRD3::NUTM1* fusion gene was discovered.

## Case report

### Patient history

**Figure 1:**
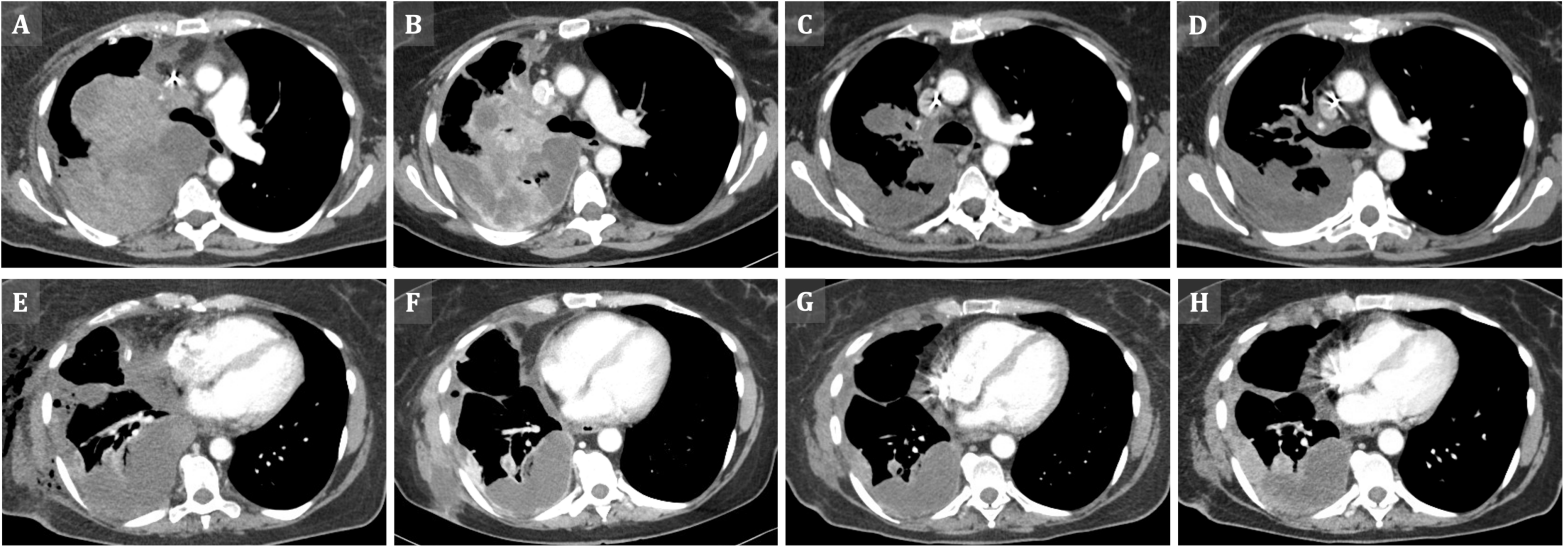
Tumor regression on sequential contrast enhanced CT scans (CECT) of the chest. **(A)** Axial chest CECT (performed on day 0, i.e. before the onset of the multimodal NUT therapy regimen) - large, highly attenuated lung mass occupying the entire right upper lung lobe with invasion of the mediastinum as well as tumoral spread to the hilar and pre-tracheal lymph nodes. **(B)** First follow-up (FU) (day 20) - increasingly heterogeneous tumor attenuation and slight shrinkage. **(C)** Second FU (day 55) - further loss in tumor attenuation (vascular supply) as well as strong volume reduction on chest CECT. **(D)** Third FU (day 77) - stable situation with respect to the residual tumor manifestations in the right lung and mediastinum. **(E)** Axial chest CECT (day 0) – right sided pleural metastases and epiphrenic tumor mass. **(F)** First FU (day 20) – volume reduction of the pleural and epiphrenic tumor manifestations. **(G)** Second FU (day 55) – stable situation. **(H)** Third FU (day 77) – slowly progredient epiphrenic and pleural tumor with increased marginal attenuation.

A 57-year-old woman with no past medical history and no history of smoking initially was evaluated for a worsening cough and dyspnea by her local specialist for internal medicine. Serial testing for COVID-19 remained negative. With a suspected pulmonary embolism, she was referred to a radiologist, who performed the initial CT scan of the chest. The CT scan revealed a large pulmonary mass (12 x 11.8 x 11 cm) in the right upper lobe with compression of the right upper lobe artery and bronchus, right-sided malignant pleural effusion with pleural metastases, enlarged ipsilateral mediastinal lymph nodes and a mediastinal shift to the left side (**Figure 1A**). Initial TNM staging for lung carcinoma was cT4 cN2 cM1a (pleural).

### Diagnosis

The patient was subsequently hospitalized in a specialized lung clinic, where pleural drainage, biopsy *via* bronchoscopy and initiation of a standard chemotherapy with carboplatin (AUC5) and paclitaxel (175 mg/m^2^) were conducted (based on a bronchoscopy guided biopsy revealing a poorly differentiated SCC, being positive for p63 and negative for TTF-1, CD56 and synaptophysin).

**Figure 2:**
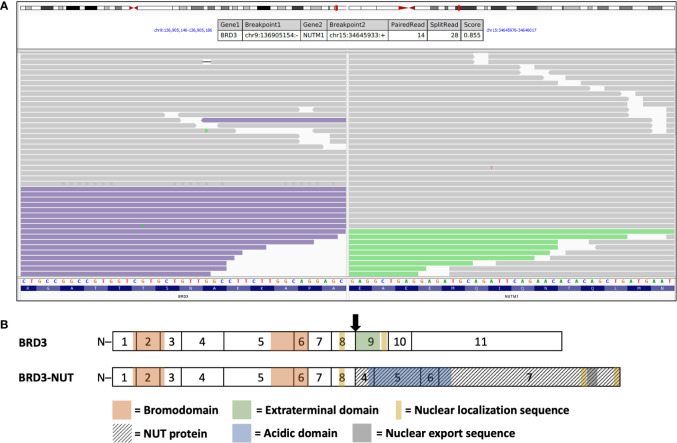
*BRD3::NUTM1* gene translocation being found in fusion RNA panel sequencing of tumor tissue. **(A)** TruSight RNA fusion panel sequencing (Illumina) revealed *BRD3* as *NUTM1* fusion partner. Integrated genome viewer split-screen view of the breakpoint regions on chromosomes 9 (left) and 15 (right). Read alignments of paired-end RNA sequencing of the identified *BRD3::NUTM1* fusion event are shown. Mate pairs mapped to the fusion reads in the *BRD3* (purple color) and *NUTM1* (green color) locus are shown. **(B)** Estimated BRD3::NUT fusion protein with breakpoints after coding exon 8 of *BRD3* (arrow; chr9:136,905,154, amino acid G549) and before exon 4 of *NUTM1* (chr15:34,645,933), resulting in the respective fusion oncoprotein. Interestingly, the extraterminal domain (BRD3, amino acids 564-641) and the second nuclear localization sequence coded on exon 9 of *BRD3* are not contained in the fusion oncoprotein.

Shortly after the first cycle of chemotherapy, she was rehospitalized with aggravated dyspnea. Subsequently, a video assisted thoracoscopy (VATS) was performed to obtain serial biopsies and to achieve a surgical pleurodesis. Reference pathology performed at the University of Erlangen, Germany, reported an undifferentiated highly malignant tumor with spindle and polygonal cells and diffuse expression of p63 and pancytokeratin, which supported the diagnosis of a non-keratinizing SCC (**Figure S1**). Additional immunostaining showed only low positivity for p40 and cytokeratin 5. The final diagnosis of NC was made *via* immunostaining with an anti-NUT antibody showing a strong nuclear signal and an additional fusion panel RNA sequencing, which revealed a *BRD3::NUTM1* gene fusion (**Figure 2** and **Figure S1**). A DNA sequencing panel for 749 genes, including standard lung-cancer diagnostics, showed no relevant alterations. At diagnosis, there was only a scattered and CD4-dominated T lymphocyte infiltration within NC tumor sites (**Figure S1**). Staining for PD-L1 showed a TPS (tumor proportion score) and CPS (combined positive score) <1% (**Figure S1**).

About three months after symptom onset, the patient was referred to the University Hospital of Tübingen, a German Comprehensive Cancer Center specialized in NC tumors, for further treatment. Here, an immediate CT scan displayed further progressive disease (PD) with additional involvement of the pleura, the diaphragm, the pericardium as well as mediastinal lymph nodes (**Figure 1E**). An additional lesion in the lateral upper region of the left mamma was identified as a NC metastasis *via* biopsy.

### Mutlimodal treatment with immunovirotherapy

**Figure 3:**
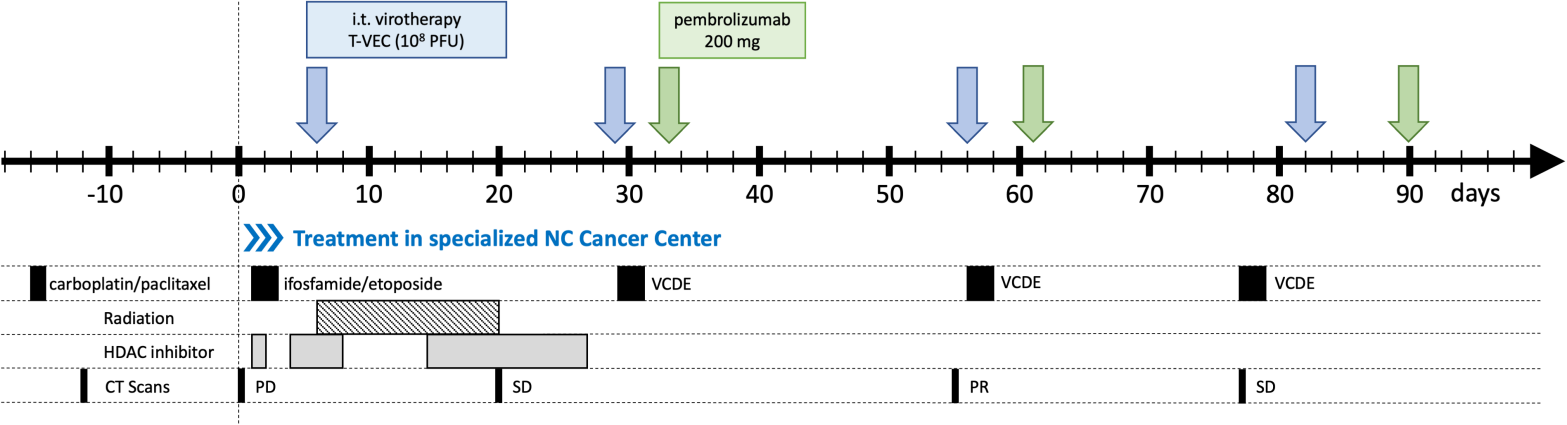
Multimodal NUT carcinoma (NC) therapy regimen, including Immunovirotherapy as a novel add-on therapy. Multimodal treatment plan. Days since first admission at specialized NC Cancer Center at University Hospital Tuebingen are shown. Mutlimodal treatment was performed with three-weekly cycles of T-VEC followed by pembrolizumab and accompanied by chemotherapy. Immunovirotherapy: T-VEC (10^8^ PFU) blue arrwos, pembrolizumab (200 mg) green arrows. Chemotherapy: CTx #1 Carboplatin/Paclitaxel (performed before referral to UKT), CTx #2 Ifosfamide/Etoposide, CTx #3 Vincristin/Cyclophosphamid/Doxorubicin/Etoposide 75%, CTx #4/#5 Vincristin/Cyclophosphamid/Doxorubicin/Etoposide 50%. Radiation: 11 x 3 Gy = 33 Gy. HDAC inhibitor: oral suberoylanilide hydroxamic acid (SAHA). PD: progressive disease, SD: stable disease, PR: partial response according to RECIST 1.1.

Shortly after admission, an intensive multimodal therapy was initiated (**Figure 3**). The patient received a second cycle of chemotherapy, now with ifosfamide (2.8 g/m^2^) and etoposide (100 mg/m^2^) followed by 11 days of radiotherapy with 3 Gy fractions each. Additionally, an oral HDAC inhibitor (suberoylanilide hydroxamic acid (SAHA), 270 mg/d) was started, but had to be discontinued shortly thereafter (before the next cycle of chemotherapy) due to a severe leukopenia and thrombopenia.

As part of our novel add-on immunovirotherapeutic concept, the first dose of T-VEC (108 PFU/ml) was administered intratumorally under ultrasound guidance on day six after the first admission (**Figure 3**, **4D**). The patient experienced only minor expected side effects (well known for any type of oncolytic virotherapy) including a slightly elevated body temperature, glazed eyes, transient fatigue and loss of appetite. The application of pembrolizumab was omitted after the first T-VEC injection to prevent enhanced initial immune clearance of the virus.

Three weeks later she received the second dose of T-VEC and a modified cycle of chemotherapy (since ifosfamide had caused severe confusion and hallucinations), following a modified VIDE protocol with a dose reduction to 75% (VCDE: vincristine 2 mg, cyclophosphamide 1.5 g/m2, doxorubicin 20 mg/m2, etoposide 150 mg/m2). Afterwards, the patient subsequently received the first dose of intravenous pembrolizumab (200 mg) as the second part of immunovirotherapy,

A CT scan after the second therapy cycle showed a partial response (PR) of the primary tumor and of pleural and pulmonal metastases as well as shrinking mediastinal lymph nodes (**Figure 1G**). These findings were accompanied by a significant clinical improvement of dyspnea and coughing with no further need for any oxygen supply.

On day 56, treatment was continued with a third intratumoral dose of T-VEC again followed by pembrolizumab. Due to prolonged pancytopenia after the second chemotherapy cycle, a further dose reduction to 50% (VCDE) was necessary. A follow-up CT scan demonstrated a generally stable disease (SD), but with a slowly progressive epiphrenic tumor mass (**Figure 1**).

**Figure 4:**
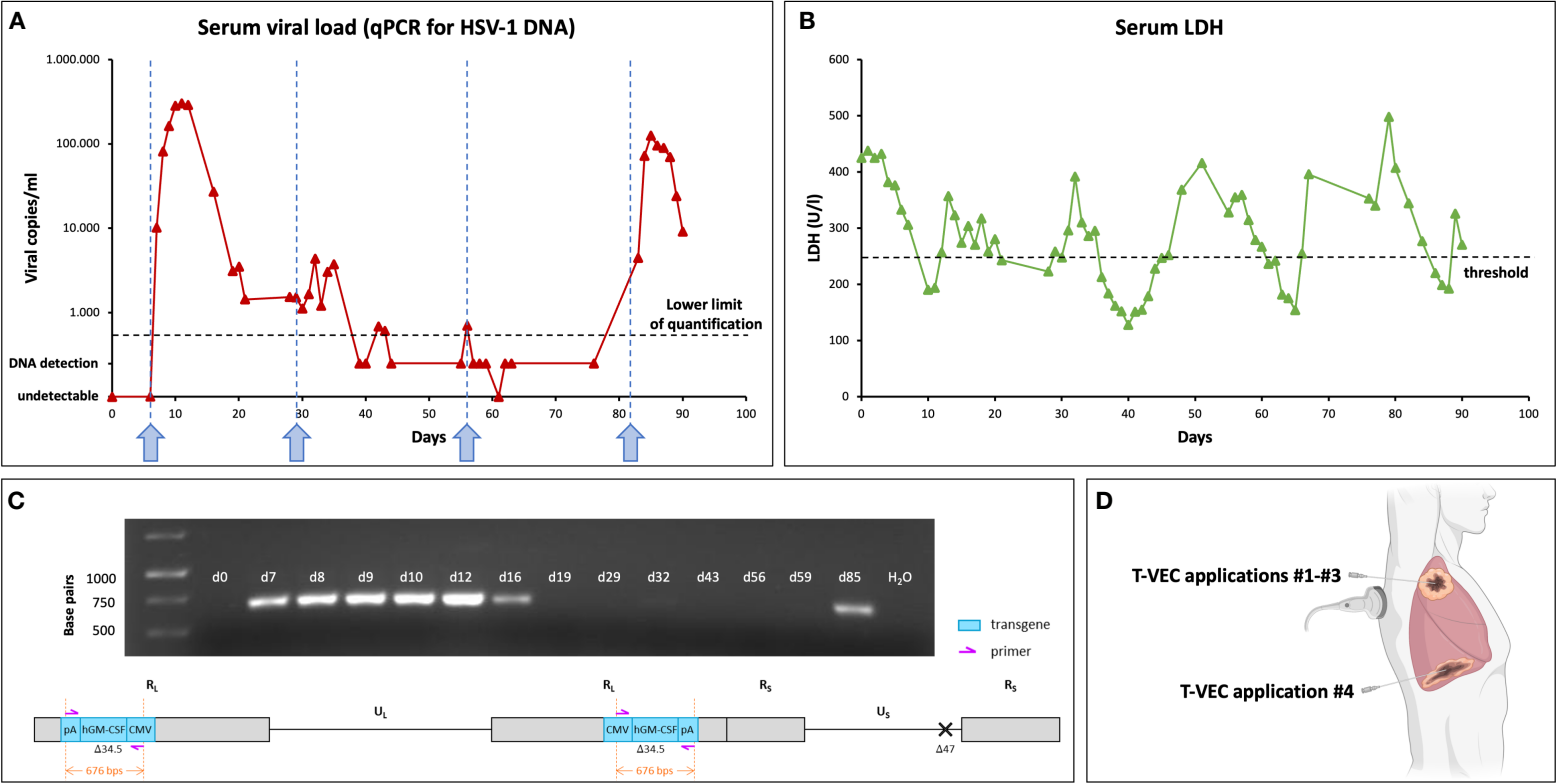
Evidence for highly efficient replication of T-VEC in NC tumor cells. **(A)** Serum viral loads over time determined by standard HSV-1 DNA real-time PCR from patient blood samples (blue arrows indicate time points of intratumoral (i.t.) injections of T-VEC). The first three times, T-VEC was administered into the right pulmonary mass identified as the primary tumor. At the time of the third i.t. injection of T-VEC, CT revealed necrosis of this pulmonary mass; as a consequence, the third injection applied again to this area no longer resulted in the production of relevant amounts of T-VEC DNA (days 56-77). Hence, injection site was changed for the fourth application of T-VEC to a highly vital and progressive epiphrenic tumor mass, now resulting again in the production of relatively high loads of HSV-1 DNA. **(B)** Serum LDH serves as a surrogate proliferation marker for NC (high levels are supposed to indicate a highly proliferative state). Each treatment cycle of the multimodal therapy regimen led to the reduction of serum LDH values below the upper normal level of 250 U/l after 5 to 10 days. However, shortly after completion of each cycle, LDH levels increased again in a highly regular manner. **(C)** Agarose gel electrophoresis of a PCR from serum samples employing T-VEC specific primers. Upper panel: Band intensities correlated well with results of standard HSV-1 qPCR **(A)**. Highest intensities were found after the first (d7-d12) and fourth (d85) T-VEC administration. Lower panel: A schematic overview of the T-VEC genome; primers (purple) bind to the flanking regions of the hGM-CSF transgene (blue) located within the terminal repeats (RL) of the unique long region (UL), being highly specific for the T-VEC genome. **(D)** The first three doses of T-VEC were administered into a tumor mass in the right upper pulmonary lobe of the lung *via* dorsal fine needle injection in the scapular line, whereas the fourth dose was injected into a right sided highly vital epiphrenic tumor manifestation at the midaxillary line. Created with BioRender.com.

After another four weeks, the fourth intratumoral T-VEC injection was administered into a highly vital right epiphrenic tumor mass, because a previous CT-Scan had revealed a necrotic primary tumor in the right upper lobe of the lung (**Figure 1D**), though slowly progressive right epiphrenic tumor manifestations (**Figure 1**). This resulted again in high serum viral loads as detected by quantitative real-time PCR for HSV-1 DNA (HSV1&2, VZV R-GENE® Kit, Biomerieux) comparable with the level obtained after the first T-VEC injection in the initial right pulmonary tumor mass (**Figure 4**). Virotherapy was again accompanied by chemotherapy (VCDE; 50%) and pembrolizumab.

Today, six months after the initial diagnosis, the patient has a stable quality of life and no need for additional oxygen supply. Therapy is still ongoing with manageable side effects.

## Discussion

This case illustrates characteristic features of the challenge to timely and correctly diagnose NC. The patient had never smoked, had no relevant past medical history and no family history of cancer. Initially, she was diagnosed with SCC of the lung. Only after the first round of SCC-oriented chemotherapy did not exhibit a proper response, immunostaining for the NUT protein was used as crucial diagnostic tool. Of note, this patient is unusual old for a NC patient, as NC is mostly diagnosed in adolescents and young adults. Suffering from thoracic NC, she is classified in the worst prognostic NC subgroup with an estimated median OS from initial cancer diagnosis of only 4.4 months.

Immunohistochemical staining with an anti-NUT antibody showed strong staining intensity which enabled correct diagnosis (**Figure S1**). Additional fusion panel RNA sequencing revealed a BRD3::NUTM1 fusion gene, generally responsible for around 15% of NC cases (**Figure 2**). Interestingly, analysis of the gene breakpoints indicated a breakpoint of BRD3 after coding exon 8 and of NUTM1 before exon 4. Accordingly, the extraterminal domain (ET) and a second nuclear localization sequence in exon 9 of the BRD3 gene are not retained in the fusion gene (**Figure 2**). Previously described fusion genes of NC with either BRD3 or *BRD4* fusions contained both bromodomains and the ET of *BRD3* or *BRD4*, thus making this the first reported case with a *BRD3::NUTM1* fusion without the ET of *BRD3* (28, 29). The ET of the BET (bromodomain and extraterminal domain) proteins is strongly conserved throughout the human BET protein family and even between different species, thus indicating an important evolutionary function. It plays a major role in recruiting transcriptional regulators such as NSD1-3 and JMJD6 (30). Our findings suggest that the protein recruiting ET is not essential for the oncogenic function of the NUT fusion protein and supports recent *in vitro* studies suggesting that the two bromodomains of BRD4 in a fusion protein with NUT are sufficient for the formation of the pathological hyperacetylated megadomains (31). However, a fully intact wild-type BRD4 protein seems to be crucial for the formation of the oncogenic nuclear complex and the pathogenesis of non-BRD4-NUT fusion NC (32–34). Importantly, the acidic domain of NUT, which is essential for downstream p300 activation, is retained in this *BRD3::NUTM1* fusion gene (35).

After no initial response to carboplatin/paclitaxel, we initiated an induction chemotherapy based on the standard VIDE protocol for Ewing Sarcoma, as these agents so far have demonstrated the best pre-clinical and case-based evidence in NC so far (6, 8, 10, 11, 36). Because of intolerable side effects (severe confusion, hallucinations) ifosfamide was exchanged with cyclophosphamide, which was shown to be non-inferior in this treatment protocol in the therapy of Ewing sarcomas (37).

The oncolytic virus T-VEC has already shown promising anti-tumoral effects in advanced sarcomas, particularly in a combinatorial treatment with the ICI pembrolizumab (38). Recently, we presented highly promising *in vitro* results exhibiting a very profound direct oncolysis in NC cell lines when being infected by T-VEC (39). Based on these results, we initiated for the first time an immunovirotherapy as a novel add-on treatment in a patient with thoracic NUT carcinoma.

Employing T-VEC, three ultrasound-guided injections into the primary tumor site were followed by a fourth injection into a metastatic epiphrenic tumor mass (**Figure 4**). Very high levels of serum viral HSV-1 DNA (up to 300,000 copies/ml) after T-VEC injection into previously untreated lesions (first and fourth injection) prove the clinical permissivness of NC for T-VEC as well as a highly efficient replication of T-VEC in NC tumors (**Figure 4**), while exhibiting only modest and easily manageable side effects. Serum HSV-1 DNA levels steeply increased after the injections and peaked 3 to 5 days after injection. Compared with experiences from melanoma treatment, higher serum viral DNA levels and a longer lasting increase in serum HSV-1 DNA after intratumoral T-VEC applications show the high susceptibility of NC to T-VEC infection and replication (40). A T-VEC-specific PCR confirmed the presence of T-VEC in serum samples and correlated with the results of standard HSV-1 qPCR (**Figure 4**).

Since replication of T-VEC in the addressed tumor sites is accompanied by a continuous release of immunostimulatory GM-CSF (being encoded in T-VEC as a transgene (39)) notable changes in the composition of the respective tumor micro-environments are triggered. A post treatment tumor specimen obtained from our NC patient at day 98 revealed (i) a partial necrosis, (ii) a switch from a CD3+CD4+ dominant to a CD3+CD8+ dominant lymphocyte infiltrate as well as (iii) an increased number of tumor infiltrating macrophages (**Figure S1** and **S2**). This indicates remarkable immunologic changes in the tumor microenvironment. Beyond that a particularly enhanced density of CD3+CD8+ lymphocytes has been found previously to be correlated with improved tumor responses to T-VEC (41).

Interestingly, lower HSV-1 serum loads were detected after injections two and three into the primary tumor site in the right upper lobe of the lung. A finding which might indicate unintended administration into mostly necrotic parts of this tumor site. Accordingly, a significant part of the injected volume of T-VEC could not induce repetitive infections of vital NC cells, ultimately causing a significantly reduced production of viral progeny. However, sequential CT scans confirmed regression and necrosis of the primary tumor site (**Figure 1**) and a stable situation in distant metastases. In summary, this implies a strong replication capacity and local anti-tumor efficacy of T-VEC in this case of thoracic NC. Further investigations are required to evaluate the effects of multimodal approaches, including such diverse treatments as chemotherapy, radiation, pleurodesis, HDAC inhibition, BET inhibition, immune checkpoint inhibition, virotherapy and possibly also other modalities.

For the fourth treatment cycle, the injection site was changed to a viable, slowly progressive epiphrenic tumor mass, which again resulted in the shedding of very high HSV-1 load levels into the patient´s circulation and pathologically confirmed necrosis (**Figure 4**; peak viral loads depicted after the fourth arrow in blue; **Figure S2**).

Additional pembrolizumab infusions were administered shortly after virotherapy to enhance the systemic anti-tumoral immune response primarily generated by T-VEC (16) (**Figure 3**). On purpose, application of pembrolizumab was not initiated right away (i.e., simultaneously with the first T-VEC injection, in order to allow unhindered T-VEC replication at this early time point and thereby induced changes in the tumor microenvironment. In this case, a clear-cut evaluation and dissection of the extent of any additional effects contributed by pembrolizumab is not possible.

During treatment, serum lactate dehydrogenase (LDH) served as a surrogate proliferation marker for NC; high levels are supposed to indicate a highly proliferative state (**Figure 4**). Each treatment cycle of the multimodal therapy regimen led to the reduction of serum LDH values below the upper normal level of 250 U/l. However, shortly after completion of each cycle, LDH levels increased again in a highly regular manner. Notably, the multimodal treatment approach employed here kept serum LDH levels in a range between 100 and 500 U/l during the whole treatment period; however, the contribution of immunovirotherapy to this aspect of disease stabilization cannot be clearly defined in this report of a single case and needs a broader investigation. This can be regarded as a therapeutic success, especially when looking at the very poor prognosis of NC cases exhibiting thoracic NC.

## Conclusion

In summary, this case demonstrates for the first time the feasibility of virotherapy in a NC patient. Treatment was well tolerated and signs of tumor response to administration of T-VEC were noted in the context of a multimodal regime. Further, tumor stabilization and improvement of the patient’s quality of life could be achieved. Furthermore, a previously unknown variant of the *BRD3::NUTM1* fusion gene was described.

## Data availability statement

The original contributions presented in the study are included in the article/**Supplementary Material**. Further inquiries can be directed to the corresponding author.

## Ethics statement

Ethical review and approval was not required for the study on human participants in accordance with the local legislation and institutional requirements. The patients/participants provided their written informed consent to participate in this study. Written informed consent was obtained from the individual(s) for the publication of any potentially identifiable images or data included in this article.

## Author contributions

LK: Conceptualization, investigation, methodology, data interpretation, writing – original draft. BC, KB, AG, SB, SG: Implementation of treatment, writing – review and editing. MH: Data interpretation, writing – original draft. SH: Investigation, methodology, data interpretation, writing – original draft. SB, JB, MC: Data interpretation, writing – review and editing. TG, SS: Implementation of treatment, writing – review and editing. AA, RS: Investigation, methodology, writing – review and editing. AH, TD, SM, FF, MR: Implementation of treatment, writing – review and editing. LZ: Supervision, writing – review and editing. UL: Supervision, conceptualization, investigation, data interpretation, implementation of treatment, writing – original draft. All authors contributed to the article and approved the submitted version.

## Funding

LK is funded by the intramural *f*ortüne program of the Faculty of Medicine, University of Tuebingen (proposal number 3007-0-0).

## Acknowledgments

We would like to thank our patient and her family. We further acknowledge support by the Open Access Publishing Fund of the University of Tübingen.

## Conflict of interest

The authors declare that the research was conducted in the absence of any commercial or financial relationships that could be construed as a potential conflict of interest.

## Publisher’s note

All claims expressed in this article are solely those of the authors and do not necessarily represent those of their affiliated organizations, or those of the publisher, the editors and the reviewers. Any product that may be evaluated in this article, or claim that may be made by its manufacturer, is not guaranteed or endorsed by the publisher.
