# Supplementary material for: Case report: Immunovirotherapy as a novel add-on treatment in a patient with thoracic NUT carcinoma
Source: Front Oncol. 2022 Oct 27;12:995744. doi: 10.3389/fonc.2022.995744 (PMC9647065; doi:10.3389/fonc.2022.995744)
Supplement: Supplementary file 1 [file DataSheet_1.pdf]

Case report: Immunovirotherapy as a novel add-on treatment in a patient with thoracic NUT carcinoma – Supplementary Material

Supplementary Figures

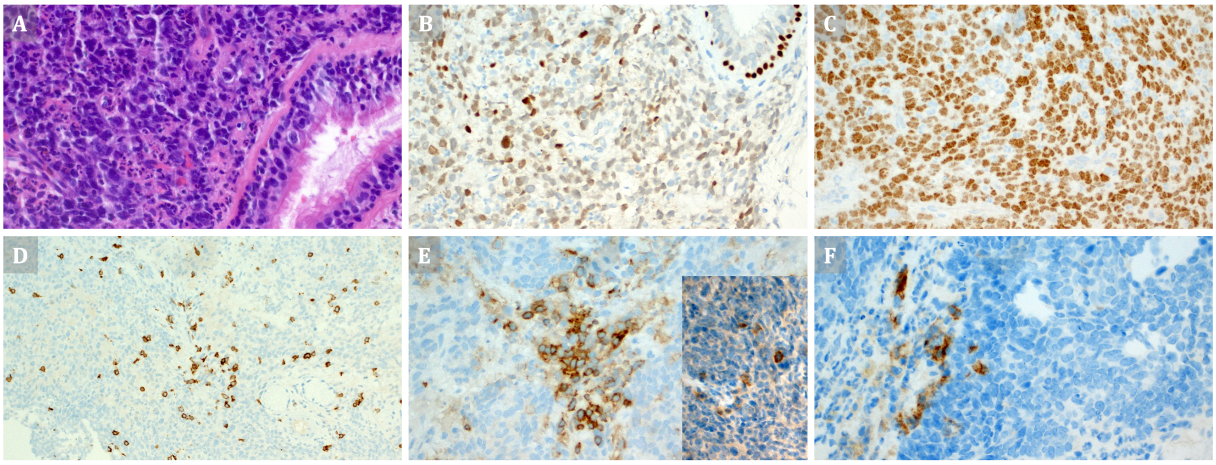

Figure S1: Initial pathological specimens obtained by video assisted thoracoscopy (VATS) showing the diagnosis of NC.

(A) Hematoxylin and eosin (H&E) staining: undifferentiated small round cell malignancy.

(B) Variable expression of p63.

(C) Strong nuclear reactivity with the anti-NUT monoclonal antibody.

(D) Scattered CD3-positive T lymphocytes.

(E) Main image: most T cells are CD4-positive. Subimage: very few T cells are CD8-positive.

(F) PD-L1 (SP263) found to be negative in the tumor cells, positive only in < 5% of immune cells (mainly located at the tumor-stromal-interphase).

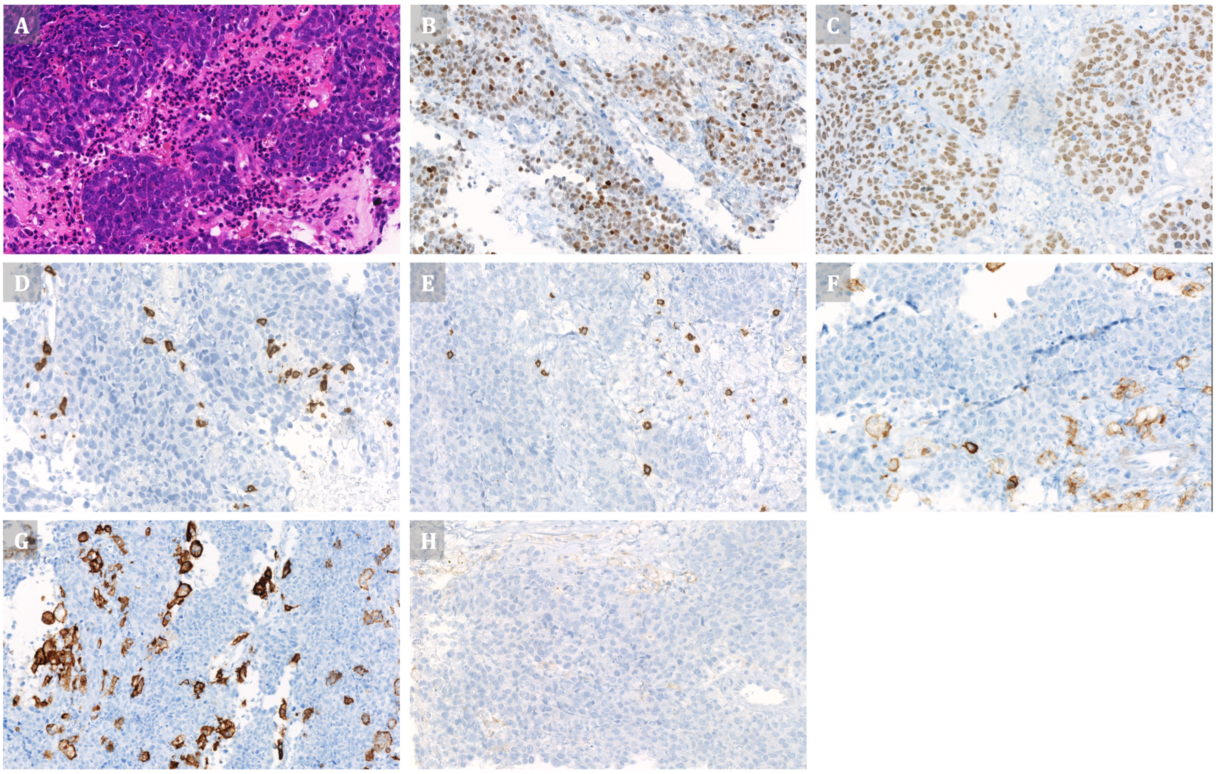

Figure S2: Post-treatment (day 98) pathological specimen of NC obtained from the right epiphrenic tumor close to the thoracic wall.

(A) H&E: undifferentiated small round cell malignancy with partial necrosis.

(B) Variable expression of p63.

(C) Strong nuclear reactivity with the anti-NUT monoclonal antibody.

(D) Scattered CD3-positive T lymphocytes.

(E) Most T cells are CD8-positive.

(F) Very few T cells are CD4 positive (larger cells with weaker staining represent macrophages, see also (G)).

(G) CD163 highlights numerous macrophages.

(H) PD-L1 (22C3) found to be positive in <1% of tumor cells and immune cells.

Methodology

Figure 2:

(A) Tumor sequencing

TruSight RNA fusion panel sequencing (Illumina, San Diego, CA, US) for 507 fusion associated genes was conducted with a tumor specimen obtained by video assisted thoracoscopy. Integrated Genome Viewer software was used for visualization.

Figure 3:

Exact mode of radiation: 33 Gy (ICRU, International commission on radiation units and measurements) in 11 fractions, single dose 3 Gy, intensity modulated radiotherapy (IMRT) and volumetric intensity modulated arc therapy (VMAT) with 6 MV photons, image guided radiotherapy (IGRT) with cone-beam computed tomography (CB-CT).

Figure 4:

(A) HSV-1 DNA real-time Polymerase Chain Reaction (PCR)

Blood serum samples were analyzed with an HSV1&2 VZV R-GENE® PCR Kit (Argene, Biomerieux, Nürtingen) targeting the US7 HSV-1 gene. The lower limit of quantification (LLOQ) was 600 viral copies/ml. Values at "DNA detection" indicate detectable HSV-1 DNA without the possibility for quantification (<600 viral copies/ml). Values at baseline indicate no detection of HSV-1 DNA.

(C) T-VEC specific PCR

Amplification of the T-VEC transgene was performed with the polymerase OneTaq (NEB) in a total reaction volume of 20 µl according to the manufacturer´s protocol, using primers binding to

the CMV promoter sequence (GGTGGGAGGTCTATATAAGC)

or the bGH-polyA sequence (ACCTTCCAGGGTCAAGGAAG)

which flank the hGM-CSF coding sequence in the T-VEC genome. The PCR product was analyzed by gel electrophoresis on a 1% agarose gel containing ethidium bromide.

(D) Created with BioRender.com.

Data sheet Figure 3/4

Days (since first admission)	HSV-1 qPCR (Copies/ml)	Immuno-virotherapy (T-VEC 10^8 PFU/ml, Pembrolizumab 200 mg)	Chermo-therapy	Radiation therapy 10*3 Gy=30 Gy	HDACi (SAHA 90 mg)	LDH (U/l)
0	0					425
1			CTx		x	438
2			CTx		x	425
3			CTx			432
4					x	382
5					x	376
6	0	Virotherapy		d1	x	333
7	10.100			d2	x	306
8	80.900			d3	x	
9	163.000			d4		
10	284.000					190
11	304.000					194
12	289.000			d5		257
13				d6		357
14				d7		323
15				d8	x	274
16	27.100			d9	x	304
17					x	270
18					x	317
19	3.090			d10	x	258
20	3.490				x	280
21	1.420				x	243
22					x	
23					x	
24					x	
25					x	
26					x	
27					x	
28	1.530					223
29	1500	Virotherapy	CTx			258
30	1.110		CTx			248
31	1.650		CTx			296
32	4.330					392
33	1.200	Pembro				310
34	3.040					286
35	3.710					295
36						213
37						184
38						162
39	<600					152
40	<600					128
41						151
42	680					155
43	602					179
44	<600					228
45						247
46						252
47						
48						368
49						
50						
51						416
52						
53						
54						
55	<600					328
56	695	Virotherapy	CTx			355
57	<600		CTx			359
58	<600		CTx			314
59	<600					279
60						267
61	0	Pembro				236
62	<600					242
63	<600					182
64						175
65						154
66						255
67						396
68						
69						
70						
71						
72						
73						
74						
75						
76	250					353
77			CTx			340
78			CTx			
79			CTx			498
80						407
81						
82		Virotherapy				344
83	4440					
84	72100					277
85	126000					
86	95000					220
87	89000					199
88	69500					192
89	23900					326
90	9120	Pembro				270
91						
92						
93						
94						
95						
96						
97						
98						
99						
100						
